# A Novel Support Vector Machine-Based Approach for Rare Variant Detection

**DOI:** 10.1371/journal.pone.0071114

**Published:** 2013-08-07

**Authors:** Yao-Hwei Fang, Yen-Feng Chiu

**Affiliations:** Division of Biostatistics and Bioinformatics, Institute of Population Health Sciences, National Health Research Institutes, Miaoli County, Taiwan, ROC; Queen’s University Belfast, United Kingdom

## Abstract

Advances in next-generation sequencing technologies have enabled the identification of multiple rare single nucleotide polymorphisms involved in diseases or traits. Several strategies for identifying rare variants that contribute to disease susceptibility have recently been proposed. An important feature of many of these statistical methods is the pooling or collapsing of multiple rare single nucleotide variants to achieve a reasonably high frequency and effect. However, if the pooled rare variants are associated with the trait in different directions, then the pooling may weaken the signal, thereby reducing its statistical power. In the present paper, we propose a backward support vector machine (BSVM)-based variant selection procedure to identify informative disease-associated rare variants. In the selection procedure, the rare variants are weighted and collapsed according to their positive or negative associations with the disease, which may be associated with common variants and rare variants with protective, deleterious, or neutral effects. This nonparametric variant selection procedure is able to account for confounding factors and can also be adopted in other regression frameworks. The results of a simulation study and a data example show that the proposed BSVM approach is more powerful than four other approaches under the considered scenarios, while maintaining valid type I errors.

## Introduction

Although common variants (CVs) that contribute to complex genetic diseases have been successfully identified from genome-wide association studies (GWAS), only a portion of heritability is explained by the identified loci. The “missing heritability” is widely believed to result from other genetic mechanisms, such as gene-gene interactions, epigenetics, and rare variants (RVs). It may be that much of the missing genetic component is due to gene variants that have relatively large effects but are too rare to be picked up by GWAS. In this case, rapid advances in next-generation sequencing technologies should enable substantial progress to be made in gene mapping. However, the statistical analysis of rare genetic variations is challenging. Because rare alleles are present in only a small number of patients, the traditional variant-by-variant approach is doomed to low power [1].

The combined multivariate and collapsing (CMC) method [2] is a pioneering statistical approach proposed for RV analysis; it tests whether the proportion of carriers of RVs is significantly different between cases and controls. Morris and Zeggini [3] expanded the CMC approach by identifying accumulations of RVs within the same functional unit via likelihood ratio association tests. Madsen and Browning [4] proposed a weighted-sum approach. In this method, variants are weighted according to their estimated frequencies in controls in a specified gene. Less-frequent variants are given a higher weight than more-common variants. The weighted-sum approach is designed for case-control studies in which a set of single nucleotide polymorphisms (SNPs) is collapsed into a single weighted average number of alleles for each individual. The Wilcoxon test is then applied to test their association with the disease.

Because the effect size of the allele may depend on its frequency, Price et al. [5] proposed a variable-threshold approach. In this method, the rare alleles are grouped together by optimizing an allele frequency threshold, which maximizes the difference between the distribution of trait values with and without rare alleles. Lin and Tang [6] proposed a general framework for detecting RVs with a weighted-sum function that covers the weights proposed by Madsen and Browning [4] and the theoretically optimal weights from estimates of regression coefficients (ERECs). They further constructed data-adaptive test statistics to combine rare mutations with opposite effects on the phenotype. Zawistowski et al. [7] proposed the cumulative minor-allele test (CMAT) derived from the standard Pearson 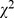 statistic. The statistical significance of CMAT is determined by permutations because the allele counts over multiple sites are not independent.

A drawback of the aforementioned methods is that they are sensitive to the presence of protective and risk variants. To account for the directions of the allele effects, Ionita-Laza et al. [8] proposed a replication-based approach. In this method, the weight of a variant is assigned relative to its observed mutant frequency in cases compared to controls. This approach is based on the calculation of two one-sided statistics, which are designed to quantify enrichment in risk and protective variants. This approach is thought to be less sensitive than other methods to the presence of a mixture of risk and protective variants. Neale et al. proposed a C-alpha statistical test for testing the presence of mixed effects across a set of RVs based on binomial distributions [9]. By testing the variance rather than the mean, their test maintains consistent power when the target set contains risk and protective variants.

The two most important features of the several recently proposed statistical methods are (1) the pooling or collapsing of multiple rare single nucleotide variants together, and (2) the application of a proper weight scheme to enrich the pooled RVs. Multiple RVs, when pooled together, collectively can have a reasonably high frequency and effect. However, if the pooled RVs are associated with the trait in different directions, then the pooling weakens the signal in associated RVs and reduces the statistical power. It is helpful to classify variants according to the directions of their effects with variants that are most likely to cause disease being up-weighted, and variants that have no effect on disease being down-weighted.

Machine learning methods have been widely applied in studies with small sample sizes [10] because statistical asymptotic properties are not applicable when sample sizes are limited or variants are rare. Support vector machine (SVM) methods have recently been found to be robust to RVs in small-sample family studies of the interactions among CVs and RVs [11]. Compared to artificial neural networks and general linear models, SVM methods have been shown to be more robust under large numbers of features for measures of model precision and accuracy [12].

The aim of the present study was to develop a new data-adaptive risk measure (RM) for identifying informative RVs. The proposed backward SVM (BSVM)-based approach considered the directions of the effects of the variants while weighting the individual RVs during the collapse. Specifically, to achieve adequate statistical power based on a reasonably high frequency and effect, all rare variants (RVs) were weighted and collapsed into either the “risk” or the “nonrisk” category; individual RVs with neutral effects were then removed backwardly from the two categories of collapsed variables in the proposed model selection procedure to retain informative RVs. Simulation studies under various scenarios and genetic mechanisms were conducted to compare the performances of the proposed BSVM and four other approaches. These approaches were applied to the type I diabetes mellitus (T1DM) dataset from Nejentsev et al. [14] for demonstration and comparison. In addition, the BSVM approach is able to account for confounding factors while detecting CVs and RVs with protective, deleterious, or neutral effects on disease.

## Materials and Methods

### SVM Method

Consider 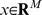 to be an M-dimension input vector. The hyperplane function 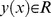 takes the form:
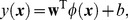
(1)where 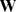 is the weight vector and *b* is the bias. 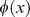 maps 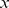 to a higher-dimension feature space, where it can be classified linearly [13]. The SVM seeks to minimize an upper bound of the generalization error by maximizing the margin 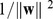 between the data and the separating hyperplane, as opposed to minimizing the empirical training error. Let *n* be the total number of individuals and 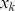 be the input vector for individual *k*. The observed affected status of individual *k*, 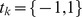, is also estimated via 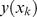 to identify factors associated with the disease. Specifically, if 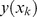 >0, then 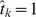 and the individual *k* is affected; otherwise, 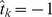 and the individual *k* is unaffected. The optimal hyperplane is obtained by solving the following quadratic form:
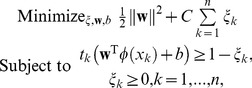
(2)where 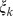 is the error term, with 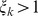 if an individual is misclassified. It follows that 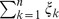 is the upper bound for the number of misclassified subjects. The constant *C* (>0) is a penalized parameter (in the present study, *C* = 10 [14]).

Next, the Lagrangian method is applied. The dual Lagrangian for the optimal hyperplane function is derived as:
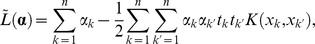
Subject to
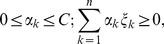
(3)where 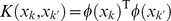 is the positive semidefinite kernel function. The kernel function used in this study is the radial basis function, 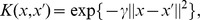 where 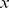 is the input vector of covariates, and 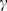 is set to 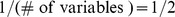, as suggested by Fan et al. [15]. The hyperplane function 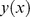 from equation (1) is estimated by
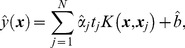
(4)where 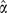 is the estimated Lagrange multiplier and 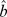 is the estimated bias. The estimated affected status 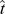 for a new vector 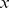 is 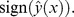 Details of the derivations are included in Appendix_S1.

### Data-driven RM

Assuming the genotype data in the studied region for 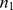 cases (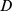) and 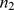 controls (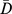) were collected. For each individual, let *H_i_* and *h_i_* be the minor (targeted) and major alleles for variant *i*, respectively, where *i* = *1*,…, *M*; *j = 1, 2*; *k_1_ = 1*,*…*, *n_1_*; *k_2_ = 1,…, n_2_*; *n = n_1_+n_2_*, and define
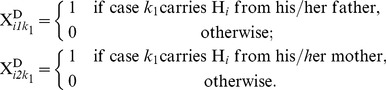
(5)


Assume that 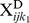 follows a Poisson distribution, with 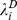 estimated by 
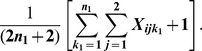
 (Madsen and Browning [4]). Accordingly, the indicator variables, 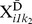 and 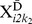, can be defined for the controls. The sums of the minor alleles of variant *i* for cases 
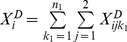
 and for controls 
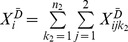
 follow the distributions of 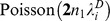 and 
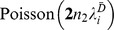
, respectively.

A variant was classified as a “risk variant” if the number of mutations in cases was larger than the number in controls, and as a “nonrisk variant” otherwise [16]. The RM of variant *i* was defined as the likelihood ratio of a mutation event in a case over a control, given the estimated mutation rate of variant *i* and its risk category. To obtain a similar measure for nonrisk variants, the RM for a nonrisk variant was defined as the likelihood ratio of a mutation event in a control over a case. If we assume that the mutation event follows a Poisson distribution, given the estimated mutation rate for a case *k_1_* or a control *k_2_*, the likelihood ratio for a mutation event of variant *i* occurring in cases (or controls) compared to controls (cases) is:
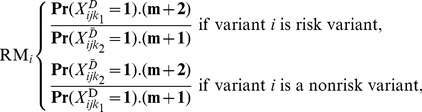
(6)where m is the total number of mutant alleles for variant *i*, and (m+2)/(m+1) is the correction factor to avoid a zero denominator, *k_1_ = 1,…, n_1_*, and *k_2_ = 1,…, n_2_*. The risk measure is proportional to the relative risk for a case (or control) having the mutant allele compared to a control (or case), which is approximately the odds ratio (OR) when the variant is rare [4], [8].

Down-weighting noncausal variants and up-weighting causal variants can improve statistical power [17]. Therefore, the risk ratio for variant *i*, denoted as RM*_i_*, was used as a weight for variant *i*. The features of RM*_i_* are as follows: (i) 1< RM*_i_* <∞; (ii) the larger the RM*_i_*, the higher the positive/negative association between variant *i* and the disease; and (iii) when there is no association between variant *i* and the disease, RM*_i_* is close to one.

### Variant Selection by the BSVM Method

Assume that there are *M* variants, including *M_1_* risk and *M_2_* nonrisk variants. Then the cumulative weighted risk minor-allele variable for individual *k*,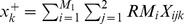, is the weighted sum from the *M_1_* risk variants. Similarly, the cumulative weighted protective minor-allele variable for individual *k*, 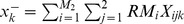, is the weighted sum from the *M_2_* nonrisk variants. We used 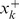 and 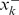 as two covariates in the kernel model in the SVM approach. Because the collapsing variant effects of these two covariates may be reduced by neutral variants, we proposed a backward variable selection method to include only informative variants, as described below.

Let R^2^ be the coefficient of determination of the model, namely:
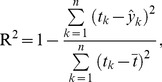
(7)where 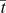 is the average of *t*, and 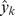 is the estimated 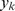 from equation (4). Although the value of R^2^ is difficult to interpret in nonlinear models, it reportedly becomes larger with a better fit and can be used to compare nonlinear models [18]. Thus, R^2^ was used as an indicator of the goodness of fit for variant selection. R^2^ approximated to 0 under the null hypothesis; and it increased when the situation moved toward the alternative hypothesis [19], [20], [21].

Starting with 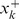 and 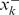, with all variants included, we denoted the two covariates for individual *k* as 
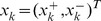
, *k = 1, …, n_1_+n_2_*. Then we performed the following steps:

1. Remove one variant (*i*) at a time from either 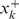 or 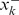. The vector containing the covariates (without the contribution from variant *i*) is denoted by:
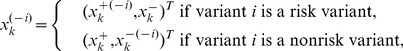
(8)where the variant 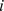 has been removed from either 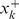 (denoted by 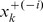) or 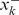 (denoted by 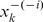).
2. Compute R^2^ when variant *i* is left out; R^2^ is denoted as R^2(−i)^.
3. Repeat steps 1 and 2 for all variants.
4. Remove variant *i* if R^2(−i)^>R^2(−i′)^ for all *i*′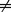 *i*, *i*′ = *1,…, M−q−1*. If R^2(−i)^≥R^2^, then substitute R^2^ by R^2(−i)^, and 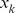 by 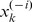, where q is the number of variants that have been removed from the backward selection procedure prior to this step.
5. Repeat steps 1–4 until R^2^>R^2(−i)^ for all *i* = 1,…, M-p, where p is the total number of “redundant” variants that have been removed from the model selection procedure prior to this step.

Next, the significance of the association between the disease and the informative variants remaining in the model was assessed by permutation tests. The empirical distribution under the null hypothesis was derived by randomly permuting each individual affected status in the absence of covariates, whereas each estimated affected status was randomly permuted in the presence of covariates [8]. The permutation was performed 1,000 times to assess the significance of individual informative variants by the SVM method.

### Simulation Study

The frequency of mutations for each variant 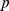 was assumed to follow Wright’s distribution [22],
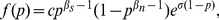
(9)where 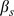 and 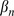 are scaled mutation rates, 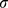 is the selection rate, and *c* is a normalizing constant. Similar to Madsen and Browning [4], 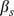 = 0.001, 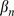 = 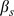/3, and 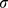 = 12. The RVs for controls were generated based on Wright’s distribution. The odds ratios (ORs) of being an affected for individual variants were estimated by the minor allele frequency (MAF) of controls (MAF_U_) and their population-attributable risks (PARs) [23]. For each variant,



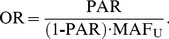
(10)The MAFs of variants among cases (MAF_A_) were determined by the ORs and MAF_U_,
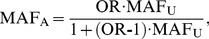
(11)and the genotypes of cases were generated accordingly [4], [8].

To mimic a real data example, a total of 183 variants with total PARs of 0.03 and 0.05 were generated. Roughly 141 of the 183 variants were RVs (MAFs <0.01) under the simulated model [4], [8]. We assumed that approximately 10 to 40 variants were the disease susceptibility variants with total PARs of 0.03 or 0.05. Two scenarios were considered: (1) equal PARs from individual variants; (2) unequal PARs from individual variants; the individual PARs followed a uniform distribution [0,1] and were renormalized to a total PAR of 0.03 or 0.05. We simulated 100 replicates for each scenario, and conducted permutations 1,000 times to obtain the empirical power and type I error. A total of 20 or 30 variants with mixture effects were generated, of which 10 variants were risk or protective variants and another 10, 20, or 30 variants were neutral. Equal numbers of cases and controls with a total sample size of 1,000 or 2,000 were simulated.

**Figure 1:**
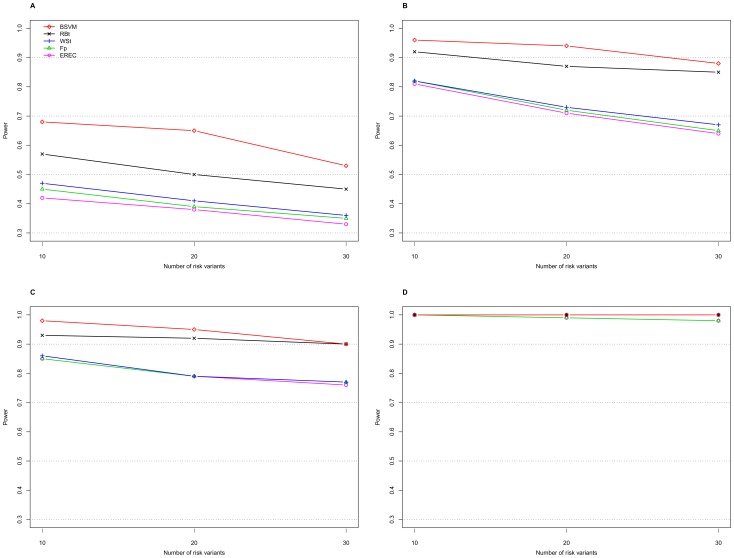
Power of the five approaches with equal PARs in the presence of different numbers of risk variants. A. PAR = 0.03 with a sample size of 1000; B. PAR = 0.05 with a sample size of 1000; C. PAR = 0.03 with a sample size of 2000; D. PAR = 0.05 with a sample size of 2000. The nominal level is 0.05.

**Figure 2:**
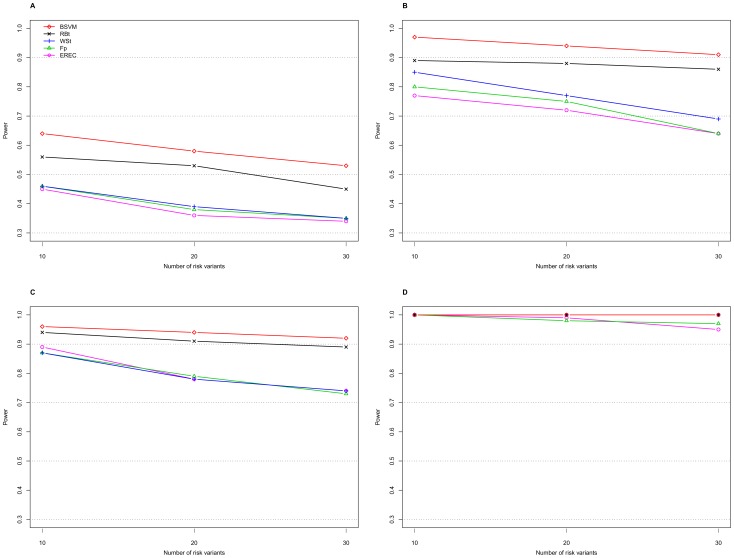
Power of the five approaches with unequal PARs in the presence of different numbers of risk variants. A. PAR = 0.03 with a sample size of 1000; B. PAR = 0.05 with a sample size of 1000; C. PAR = 0.03 with a sample size of 2000; D. PAR = 0.05 with a sample size of 2000. The nominal level is 0.05.

**Figure 3:**
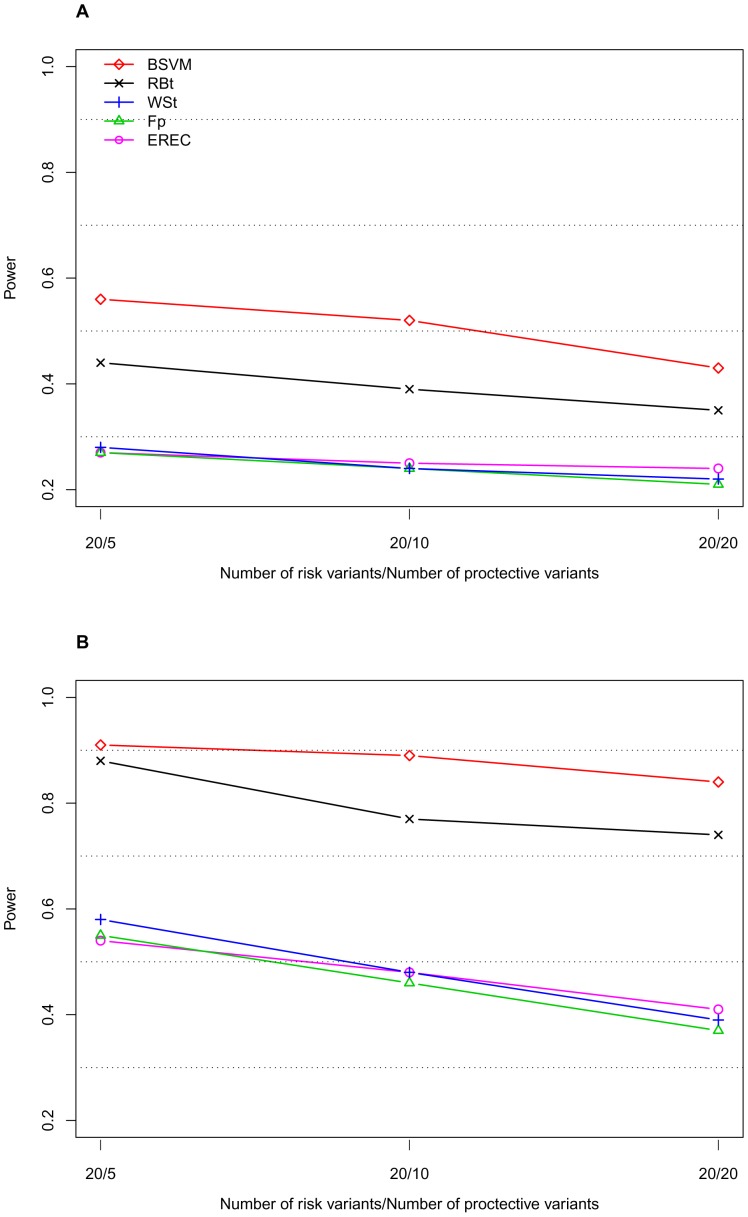
Power of the five approaches with equal PARs in the presence of a mixture of risk, neutral, and protective variants. The sample size is 1000. The nominal level is 0.05. A. PAR = 0.03; B. PAR = 0.05.

Table 1 displays the type I error and power results of the proposed BSVM method obtained with the proposed RM weighting and the weight functions from four different methods: namely, the weighted-sum test (WSt) [4], the replication-based test (RBt) [8], score tests with the weight functions based on frequency estimates in the pooled sample (Fp), and the EREC proposed by Lin and Tang [6]. Comparisons between Fp, EREC, C-alpha [9], the SNP-set Kernel Association Test (SKAT) [17], and the Summed Score Test (SSU) [24] were extensively studied by Lin and Tang [6]; therefore, these comparisons are not illustrated here. We considered the genetic mechanism with risk and neutral RVs in the absence or presence of protective variants; each risk variant had an equal PAR (Table 1). Alternatively, we compared the proposed methods with the other four approaches in terms of the type I error (Table 2) and power in the absence (Figures 1 and 2) or presence (Figure 3) of protective variants with an equal or unequal PAR. Additionally, the significance levels of individual variants were computed for the 10 individual risk variants out of 183 variants (Table 3).

**Table 1:** Type I error and power of the proposed BSVM approach, based on five weighting schemes at a nominal level of 0.05.

		Without Protective Variants			With Protective Variants		
WeightingScheme	PAR	No. of Variants Risk/Protective/Neutral	Type I error	Power	No. of Variants Risk/Protective/Neutral	Type I error	Power
RM	0	0/0/20	0.05	–	0/0/30	0.05	–
	0.03	10/0/10	–	0.76	10/10/10	–	0.66
	0.05	10/0/10	–	0.99	10/10/10	–	1
RBt	0	0/0/20	0.08	–	0/0/30	0.09	–
	0.03	10/0/10	–	0.72	10/10/10	–	0.63
	0.05	10/0/10	–	0.97	10/10/10	–	0.99
WSt	0	0/0/20	0.07	–	0/0/30	0.07	–
	0.03	10/0/10	–	0.69	10/10/10	–	0.53
	0.05	10/0/10	–	0.93	10/10/10	–	0.93
Fp	0	0/0/20	0.02	–	0/0/30	0.03	–
	0.03	10/0/10	–	0.68	10/10/10	–	0.55
	0.05	10/0/10	–	0.88	10/10/10	–	0.88
EREC	0	0/0/20	0.10	–	0/0/30	0.1	–
	0.03	10/0/10	–	0.85	10/10/10	–	0.68
	0.05	10/0/10	–	1	10/10/10	–	1

Weighting schema are: RM, risk measure (present study); RBt, replication-based test; WSt, weighted-sum test; Fp, score test with the weight function based on frequency estimates in the pooled sample; and EREC, score test with the weight function based on the EREC proposed by Lin and Tang. PAR, population-attributable risk.

**Table 2:** Type I error for the five approaches.

N	NominalLevel	Fp	EREC	WSt	RBt	BSVM
1000	0.05	0.05	0.05	0.05	0.05	0.06
	0.025	0.02	0.02	0.03	0.02	0.02
	0.01	0.01	0.01	0.01	0.01	0.01
2000	0.05	0.05	0.05	0.05	0.05	0.06
	0.025	0.02	0.02	0.03	0.03	0.03
	0.01	0.01	0.01	0.01	0.01	0.01

**Table 3:** Identification of ten significant individual risk variants (*P*≤0.05) out of 183 rare variants using the SVM method.

Variant ID	Avg. *P*-value	Variant ID	Avg. *P*-value
PAR = 0.03		PAR = 0.05	
Variant 1	0.02799	Variant 1	0.00841
Variant 2	0.00510	Variant 2	0.00351
Variant 3	0.03591	Variant 3	0.00108
Variant 4	0.02532	Variant 4	0.00071
Variant 5	0.0259	Variant 5	0.00108
Variant 6	0.03815	Variant 6	0.01925
Variant 7	0.04197	Variant 7	0.02854
Variant 8	0.0151	Variant 8	0.0083
Variant 9	0.02398	Variant 9	0.02105
Variant 10	0.00471	Variant 10	0.02978

## Results

The proposed RM weighting with the BSVM method had the highest power among the five weightings under scenarios with risk variants or a mixture of risk and protective variants (Table 1). Table 2 presents the type I errors from the five approaches. Three nominal levels (0.05, 0.025, and 0.01) were examined under the null hypothesis. The type I errors of the five methods were all consistent with the nominal levels.

Figures 1 and 2 show the powers of the five approaches under scenarios with a total PAR of 0.03 or 0.05 equally or unequally contributed from 10, 20, or 30 risk variants. For the same total PAR, the power decreased when the contributions from individual risk variants decreased. The performances of the Fp, EREC, and WSt methods were compatible, whereas the RBt and BSVM approaches consistently had higher powers than the other three methods. In particular, the proposed BSVM method outperformed other approaches in power under all of the scenarios considered. When the sample size was 2,000 with a PAR 0.05, the powers for all of the approaches were close to 1 (Figures 1 and 2).

It is likely that the studied region, which involves multiple genes under different pathways, contains risk and protective variants. Therefore, we studied the performance of the five approaches with a mixture of risk and protective variants in the studied region (Figure 3). The total PAR from the 20 risk variants was 0.03 or 0.05. Each risk variant had an equal per-variant PAR (i.e., total PAR divided by 20); and each protective variant had the same per-variant PAR (i.e., total PAR divided by 20). We generated 1,000 samples for each scenario.

Compared to the scenario with 30 risk variants, in the presence of risk and protective variants, the power decreased from 0.35 to 0.24 (*−*11%) for Fp, from 0.33 to 0.25 (*−*8%) for EREC, from 0.36 to 0.24 (*−*12%) for WSt, from 0.45 to 0.39 (*−*6%) for RBt, and from 0.53 to 0.52 (*−*1%) for BSVM. These results suggest that the BSVM method was more robust in the scenario with a mixture of risk and protective variants than the other four methods. The BSVM method further identified informative variants with significant effects on disease (Table 3). The significance reflects the magnitude of an effect (the stronger the effect, the greater its significance).

### Application to the Study of T1DM

We applied five approaches – WSt, RBt, Fp, EREC, and BSVM – to the T1DM study by Nejentsev et al. [25]. These authors resequenced the exons and splice sites of 10 candidate genes in 480 cases and 480 controls. We reanalyzed this dataset using the WSt, RBt, Fp, EREC, and BSVM approaches. We generated 100 replicates of 480 cases and 480 controls with 10 genes, using the resampling technique based on the supplemental file provided by Nejentsev et al. [25]. This procedure was repeated for 100 replicates with 10,000 permutations to obtain the average P-values. For each replicate, the observed frequency for each variant in the original study was fixed. The RVs were assumed to be independent from each other because of their rareness.

The *IFIH1* gene was identified by all five approaches. The *CLEC16A* gene showed modest significance in all approaches, except for the Fp method (Table 4). The BSVM method showed the most significant levels for these two genes (*P* = 0.000006 for *IFIH1*, and *P* = 0.008107 for *CLEC16A*). These findings are consistent with the results from Iuliana et al. [8]. We tried to identify the significance of individual variants associated with T1DM in the *IFIH1* gene using the SVM method (Table 5). The significant RVs were rs35667974 (*P* = 0.0049 by the exact test, *P* = 0.007 by SVM) and rs35337543 (*P* = 0.000044 by the exact test, *P*<0.0001 by SVM); and the significant CV was rs1990760 (*P* = 0.00086 by the chi-squared test, *P* = 0.0025 by SVM).

**Table 4:** Significance of the T1DM genes from the five methods.

		Average P-value				
Gene	#SNVs*	Fp	EREC	WSt	R	BSVM
*IFIH1*	29	0.00494	0.00001	0.00028	0.00013	0.000006
*CLEC16A*	45	0.24275	0.01101	0.02347	0.01548	0.008107

* SNVs: single nucleotide variants.

**Table 5:** Association analysis of four significant variants in *IFIH1* gene from T1DM patients and controls.

rs# or ss#(for new SNPs)	Location	Major allele	Minor allele	T1DM ChMA§	Controls ChMA	*P*-value	*P*-value
Rare						SVM	Exact test
rs35667974	exon 14, I923V	A	g	7/960	23/960	0.007	0.0049
rs35337543	intron 8, +1splice	G	c	3/960	24/960	<0.0001	0.000044
ss107794690	exon 11, T702I	C	t	1/960	4/960	0.0716	0.37
ss119336617	exon 2,N160D	A	g	0/960	2/960	0.2495	0.5
Common						SVM	*x* ^2^
rs1990760	exon 15, T946A	A	g	298/960	367/960	0.0025	0.00086
rs3747517	exon 13, A843H	G	a	241/960	252/960	0.8069	0.58

§ ChMA, estimated fraction of chromosomes with minor alleles.

## Discussion

We have proposed a novel data-adaptive BSVM-based selection procedure to identify a region with RVs associated with complex traits and individual variants in the disease-associated gene/region. Likelihood ratios of the Poisson distributions for individual variants between cases and controls were used to weight the variants, which were collapsed into two variables according to the effect directions. The selection procedure was applied to the two collapsed variables to select informative variants associated with disease. Permutation tests were used to assess the significance of the gene/region with selected variants and to identify possible functional individual variants in the significance region.

This approach has several useful features. As an SVM approach, it does not rely on asymptotic statistical theory and is applicable to studies with limited sample sizes. By starting with collapsed high-frequency variants, it eases the concern of low power due to the sparseness of RVs. The data-adaptive weighting scheme accounts for the directions of effects of risk and nonrisk variants. This RM-based weighting and model selection procedure could be adopted in other approaches for identification of RV. The categorized, collapsed variables are nearly orthogonal to each other, which improves the power for identifying informative variants in the presence of mixed effects. This nonparametric approach does not require any prespecified model assumptions, can be applied to scenarios with CVs and RVs by including CVs as additional covariates, and allows for covariate adjustments. Finally, the approach can be used to identify significant individual variants associated with disease for further study, diagnostics, and predication.

In the simulation study and data example, the proposed method outperformed other current methods in terms of power, while maintaining valid type I errors. The choice of C (penalty) in equation (2) is a trade-off between precision and variation. Previous studies have shown that the performance for RV prediction is similar when C is set at 1, 10, or 100 [14]. Therefore, we set C at 10. The value of the parameter gamma (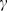) in the radial basis kernel function suggested by Fan et al. [15] appeared to be more robust and powerful than other options in the simulation study (data not shown). Instead of being prespecified, this parameter could alternatively be estimated via a grid search, although this process is time consuming [26].

In the data example, BSVM appeared to be the most powerful of the five methods for RV analysis; but it also took the longest time to run [11]. When there are numerous markers (e.g., from GWAS or whole-genome sequencing data), applying SNP set-, gene-, or pathway-based analysis in SVM-based methods can substantially reduce the computing time. The SVM approach has been shown to be robust to family structure and population stratification in population-based studies [11]. Therefore, the performance of the proposed method and its extension to family studies warrant further investigation. The computing programs (written in MATLAB) for generating the example data and the proposed method are available online at http://sb.nhri.org.tw/BSVM/.

## Supporting Information

Appendix S1 **Technical details of the SVM Method.**
(DOC)
