# Supplementary material for: A Novel Support Vector Machine-Based Approach for Rare Variant Detection
Source: PLoS One. 2013 Aug 7;8(8):e71114. doi: 10.1371/journal.pone.0071114 (PMC3737136; doi:10.1371/journal.pone.0071114)
Supplement: Appendix S1 — Technical details of the SVM Method. (DOC) [file pone.0071114.s001.doc]

Technical details of the SVM Method

Because there is no analytical method to determine an appropriate in equation (1) to solve this quadratic programming problem, Lagrange dual function and multipliers (denoted by $\alpha_{j}$ and $\beta_{j}$) are used to solve this problem [13]. To minimize the objection function with the constraints in equation (2), we use the Lagrangian:

$L\left(\mathbf{w},b,\alpha_{j}\right)=\frac{1}{2}\left\|\mathbf{w}\right\|^{2}+C\sum_{j=1}^{N} \xi_{j}-\sum_{j=1}^{N} \alpha_{j}\left(t_{j}y\left(\mathbf{x}_{j}\right)-1+\xi_{j}\right)-\sum_{j=1}^{N} \beta_{j}\xi_{j},$ (12)

where {$\alpha_{j}\geq0$} and {$\beta_{j}\geq0$} are Lagrange multipliers. The corresponding set of Karush-Kuhn-Tucker (KKT) conditions are given by $\alpha_{j}\geq0$, $t_{j}y\left(x_{j}\right)-1+\xi_{j}\geq0$, $\alpha_{j}\left(t_{j}y\left(x_{j}\right)-1+\xi_{j}\right)=0$, $\beta_{j}\geq0$, $\xi_{j}\geq0$, and $\beta_{j}\xi_{j}=0$, where *n* = 1,…, *N*. Then, we optimize **w**, *b*, and {$\xi_{j}$}, making use of the definition (2) of$y\left(x_{j}\right)$, to obtain:

(13)

$\frac{\partial L}{\partial b}=0\text{ }\Rightarrow\text{ }\sum_{j=1}^{N} \alpha_{j}t_{j}=0,$ (14)

$\frac{\partial L}{\partial\xi_{j}}=0\text{ }\Rightarrow\text{ }\alpha_{j}=C-\beta_{j}.$ (15)

Using these results to eliminate **w**, *b*, and {$\xi_{j}$} from the Lagrangian, we obtain the dual Lagrangian in the form

$\tilde{L}\left(\mathbf{\alpha}\right)=\sum_{j=1}^{N} \alpha_{j}-\frac{1}{2}\sum_{j=1}^{N} \sum_{k=1}^{N} \alpha_{j}\alpha_{k}t_{j}t_{k}k\left(x_{j},\text{ }x_{k}\right),$ (16)

where is the positive semidefinite kernel function.

To determine the constraints, we note that $\alpha_{j}\geq0$ is required because we are dealing with Lagrange multipliers. Equation (15) together with $\beta_{j}\geq0$ implies $\alpha_{j}\leq C$. Therefore, equation (16) must be minimized with respect to the dual variables {$\alpha_{j}$}, subject to

$0\leq\alpha_{j}\leq C,$ (17)

$\sum_{j=1}^{N} \alpha_{j}\xi_{j}\geq0,$ (18)

for *n* = 1,…, *N*, where equation (17) is known as the box constraint. This situation again represents a quadratic programming problem. If we substitute equation (13) into equation (1), we obtain predictions for new data points, by using

$y\left(x\right)=\sum_{j=1}^{N} \alpha_{j}t_{j}k\left(x,\text{ }x_{j}\right)\text{ +}b$. (19)

We can now interpret the resulting solution. As before, a subset of the data points may have $\alpha_{j}=0$, in which case they do not contribute to the predictive model (19). The remaining data points constitute the support vectors. These data have $\alpha_{j}>0$. Hence, from $\alpha_{j}\left(t_{j}y\left(x_{j}\right)-1+\xi_{j}\right)=0$, they must satisfy

$t_{j}y\left(x_{j}\right)=1-\xi_{j}.$ (20)

If $\alpha_{j}<C$, then equation (15) implies that $\beta_{j}>0$, which from $\beta_{j}\xi_{j}\geq0$ requires $\xi_{j}=0$ and, hence, such points lie on the margin. Points with $\alpha_{j}=C$ lie inside the margin and are either correctly classified if $\xi_{j}\leq0$ or misclassified if $\xi_{j}>1$.

To determine the parameter *b* in equation (1), we note that those support vectors for which $0<\alpha_{j}<C$ have $\xi_{j}=0$, such that $t_{j}y\left(x_{j}\right)=1$ and, hence, will satisfy

$t_{j}\left(\sum_{k\in SV} \alpha_{k}t_{k}k\left(x_{j},\text{ }x_{k}\right)\text{+}b\right)=1.$ (21)

Again, a numerically stable solution is obtained by averaging, yielding:

$b=\frac{1}{N_{M}}\sum_{j\in M} \left(t_{j}-\sum_{k\in S.V} \alpha_{k}t_{k}k\left(x_{j},\text{ }x_{k}\right)\right),$ (22)

where *M* denotes the set of indices of data points having $0<\alpha_{j}<C$.

The positive semidefinite kernel function,, which can be substituted for a nonlinear feature-space transformation. For example, the commonly used radial-basis function kernel [27] is defined as:

$k\left(x_{j},x_{k}\right)=\exp\left(-\gamma\left\|x_{j}-x_{k}\right\|^{2}\right),$ (23)

where $\gamma$ is a kernel parameter, $1\leq i,j\leq N$. Thus, the estimated target value $\text{t}$ for a given new
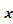
 is obtained as follows:

$$\text{t=sign(}y\left(x\right)\text{)=sign}\left(\sum_{j=1}^{N} \alpha_{j}t_{j}k\left(x,\text{ }x_{j}\right)\text{ +}b\right).$$
